# Pulmonary lymphangitic carcinomatosis from gallbladder cancer mimicking diffuse alveolar haemorrhage

**DOI:** 10.1002/rcr2.540

**Published:** 2020-02-17

**Authors:** Hisao Higo, Noriyuki Suzaki, Takuya Nagata, Taro Togami, Nobuya Ohara, Masaomi Marukawa

**Affiliations:** ^1^ Department of Internal Medicine Kagawa Rosai Hospital Marugame Japan; ^2^ Department of Radiology Kagawa Rosai Hospital Marugame Japan; ^3^ Department of Pathology Kagawa Rosai Hospital Marugame Japan

**Keywords:** Diffuse alveolar haemorrhage, gallbladder cancer, haemoptysis, pulmonary lymphangitic carcinomatosis

## Abstract

Diagnosis in cases with pulmonary lymphangitic carcinomatosis as a primary manifestation is difficult due to unawareness of the cancer. An 81‐year‐old man was admitted due to a one‐week history of dyspnoea and haemoptysis. Chest computed tomography showed diffuse bilateral ground‐grass opacity and partial consolidation. We suspected diffuse alveolar haemorrhage. High‐dose methylprednisolone and cyclophosphamide did not improve his condition and he died from respiratory failure. Autopsy revealed pulmonary lymphangitic carcinomatosis of whole lungs and primary gallbladder cancer. We should consider pulmonary lymphangitic carcinomatosis in the differential diagnosis of patients with haemoptysis and diffuse lung opacity of unknown origin.

## Graphical abstract

An 81‐year‐old man was admitted due to dyspnoea and haemoptysis. We suspected diffuse alveolar haemorrhage based on computed tomography (CT) findings of diffuse bilateral ground‐grass opacity and partial consolidation. Autopsy revealed pulmonary lymphangitic carcinomatosis of whole lungs and primary gallbladder cancer.

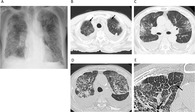

## Introduction

Pulmonary lymphangitic carcinomatosis is caused by lymphatic spreading of metastatic cancer cells in the lungs. Most patients present with subacute, progressive dyspnoea. A previous study reported an incidence of lung metastases of 6–8% in patients with cancer 1. We should consider lymphangitic carcinomatosis as one of the possible diagnoses in case of progressive dyspnoea complaints in patients with cancer. However, sometimes, the diagnosis is difficult when the primary manifestation is pulmonary lymphangitic carcinomatosis, because we are unaware of the cancer.

## Case Report

An 81‐year‐old Japanese man was admitted to our hospital due to haemoptysis and dyspnoea lasting for a week. He had occupational history of asbestos exposure.

**Figure 1:**
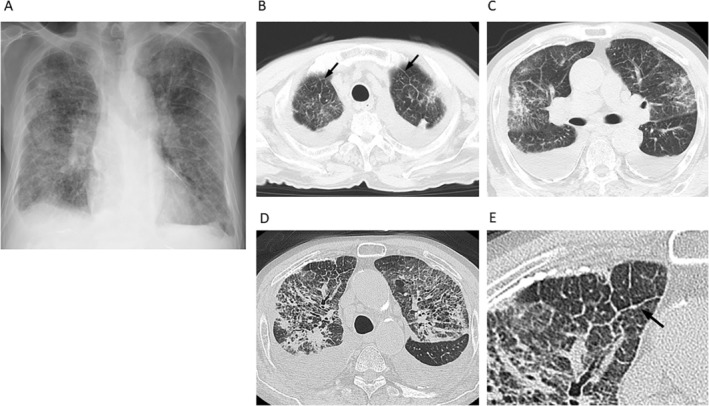
Chest X‐ray and computed tomography (CT) on admission. (A) Chest X‐ray showed bilateral ground‐grass opacity, consolidation, and blunting of the bilateral costophrenic sulci. (B, C) Chest CT revealed bilateral ground‐grass opacity and partial consolidation with upper lobe predominance. Smooth thickened interlobular septa were seen only in the bilateral lung apexes (arrows). (D, E) High‐resolution CT on hospital day 7. The thickened interlobular septa were slightly irregular (arrow).

On admission, his body temperature was 37.2°C and O_2_ saturation was 86%. Bilateral fine crackles were heard on chest auscultation. Chest X‐ray showed diffuse bilateral ground‐grass opacity and consolidation and blunting of the bilateral costophrenic sulci (Fig. 1A). Chest computed tomography (CT) revealed diffuse bilateral ground‐grass opacity and partial consolidation with upper lobe predominance (Fig. 1B, C). Smooth thickened interlobular septa were seen only in the bilateral lung apexes (Fig. 1B). Mediastinal lymphadenopathy, bilateral pleural effusions, and pleural plaques were also observed. Laboratory findings demonstrated leucocytosis (9100/μL) and elevated serum C‐reactive protein level (4.2 mg/dL), while platelet count, prothrombin time‐international normalized ratio, and activated partial thromboplastin time were normal. Serum anti‐dsDNA and anti‐Sm antibody levels were elevated (40.2 and 20.4 IU/mL, respectively), but there were no physical findings suggesting systemic lupus erythematosus. Antineutrophil cytoplasmic autoantibodies and anti‐glomerular basement membrane antibody were negative. Thoracentesis revealed that the pleural fluid was exudative, with lymphocytic predominance (93%). Cytology of the pleural fluid was negative. Tazobactam/piperacillin was administered from the first hospital day, but with no effect. On the basis of the chest CT findings and the persisting haemoptysis, we suspected diffuse alveolar haemorrhage. Bronchoscopy was not performed because his consent was not obtained due to severe dyspnoea. Methylprednisolone 1 g/day intravenously for three days followed by prednisolone 0.5 mg/kg was administered from the second hospital day. However, haemoptysis and dyspnoea were not improved. High‐resolution CT (HRCT) was performed on the hospital day 7. The area of ground‐grass opacity and consolidation was expanded and the thickened interlobular septa were slightly irregular (Fig. 1D, E). We repeated high‐dose methylprednisolone therapy as described above and added cyclophosphamide on hospital day 9. Despite the treatment, the patient's condition did not improve and he died of respiratory failure on hospital day 21.

**Figure 2:**
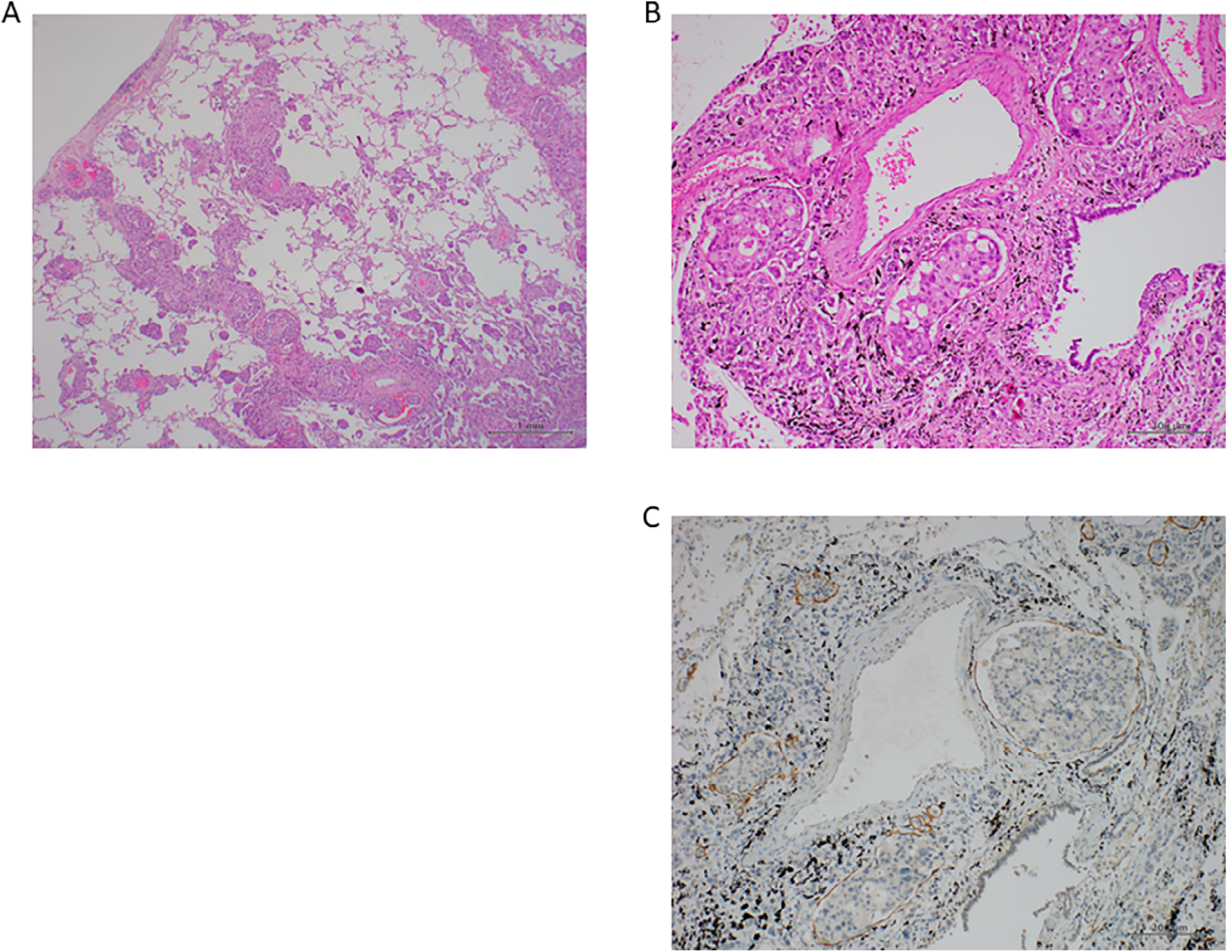
Histological autopsy findings. (A, B) Marked cancer metastasis with vessel invasion and pulmonary lymphangitic carcinomatosis of the lungs were seen (haematoxylin and eosin staining). Scale bar = 1 mm and 20 μm. (C) Immunohistochemistry of D2‐40, which detects lymphatic vessels, confirmed that the lymphatic vessels were filled with cancer cells. Scale bar = 20 μm.

Autopsy revealed marked cancer metastases with vessel invasion and pulmonary lymphangitic carcinomatosis of the whole lungs (Fig. 2). There were no findings suggesting vasculitis or alveolar haemorrhage. Poorly differentiated adenocarcinoma was found in the gallbladder, which had infiltrated the hepatic hilar region and the right liver lobe. Infiltration of the pancreas, duodenum, and stomach, and cancer metastasis to the bilateral adrenal grand were also seen. A final diagnosis of pulmonary lymphangitic carcinomatosis from gallbladder cancer was made.

## Discussion

Although rarely, cancer may present with pulmonary lymphangitic carcinomatosis as a primary manifestation. If an extrathoracic organ is the primary site, there is a possibility of misdiagnosis due to unawareness of the cancer. There have been reports on such misdiagnosed cases mimicking interstitial lung disease 2, sarcoidosis 3, and military tuberculosis 4. The most common complaint in these patients was cough or dyspnoea, whereas in our case, the patient had continuous haemoptysis and was misdiagnosed with diffuse alveolar haemorrhage.

This case had some features similar to those of diffuse alveolar haemorrhage. The patient had typical symptoms, haemoptysis and dyspnoea, with acute onset 5, 6. Bilateral fine crackles were heard on chest auscultation, which is also compatible with the disease 7. The most common CT findings of diffuse alveolar haemorrhage are patchy ground‐glass opacities and consolidation on the bilateral lungs 8. Thickening of the intralobular septa is also sometimes seen in diffuse alveolar haemorrhage 9. All these features were noted in the present case. In addition, serum antibodies suggesting systemic lupus erythematosus, which could induce diffuse alveolar haemorrhage, were positive.

On the other hand, there were features that made it difficult to diagnose pulmonary lymphangitic carcinomatosis. First, haemoptysis is a relatively rare symptom in pulmonary lymphangitic carcinomatosis. Common symptoms of this disease are dyspnoea and cough 10. Second, pleural fluid cytology was negative. Pleural dissemination was not seen on autopsy, but the cause of pleural effusion was thought to be increased pleural permeability or decreased lymphatic flow due to the cancer 11. Third, although the origin site of the pulmonary lymphangitic carcinomatosis was the gallbladder, the patient did not have any symptoms related to the primary disease. The most common complaints in patients with gallbladder cancer are pain under the right ribs, weight loss, anorexia, nausea, and vomiting 12.

For these reasons, it was difficult to make a correct diagnosis. However, we should have suspected pulmonary lymphangitic carcinomatosis because HRCT revealed slightly irregular thickness of the interlobular septa, although conventional CT did not detect the irregularity. Irregular thickness of the interlobular septa is a relatively specific feature of lymphangitic carcinomatosis 10, 13. If bronchoscopy could have been performed, we might have reached a correct diagnosis. Bronchoalveolar lavage was reported to be useful in the diagnosis of pulmonary lymphangitic carcinomatosis 14, 15 as well as diffuse alveolar haemorrhage 9, 16.

In conclusion, we reported a rare case of pulmonary lymphangitic carcinomatosis mimicking diffuse alveolar haemorrhage. To the best of our knowledge, this is the first report of pulmonary lymphangitic carcinomatosis as the first manifestation of gallbladder cancer. We should consider pulmonary lymphangitic carcinomatosis in the differential diagnosis when encountering patients with haemoptysis and diffuse lung opacity of unknown origin, even in cases without past history of cancer.

### Disclosure Statement

Appropriate written informed consent was obtained for publication of this case report and accompanying images.

## References

[1] Bruce DM , Heys SD , and Eremin O . 1996 Lymphangitis carcinomatosa: a literature review. J. R. Coll. Surg. Edinb. 41:7–13. 8930034

[2] Gilchrist FJ , Alton H , Brundler MA , et al. 2011 Pulmonary lymphangitic carcinomatosis presenting as severe interstitial lung disease in a 15‐year‐old female. Eur. Respir. Rev. 20:208–210. 21881149 10.1183/09059180.00000911 PMC9584110

[3] Thomas A , and Lenox R . 2008 Pulmonary lymphangitic carcinomatosis as a primary manifestation of colon cancer in a young adult. CMAJ 179:338–340. 18695182 10.1503/cmaj.080142 PMC2492966

[4] Welch J , and Welsh G . 2008 Lymphangitis carcinomatosis mimicking miliary tuberculosis. N. Z. Med. J. 121:123–125. 19079445

[5] Primack SL , Miller RR , and Muller NL . 1995 Diffuse pulmonary hemorrhage: clinical, pathologic, and imaging features. AJR Am. J. Roentgenol. 164:295–300. 7839958 10.2214/ajr.164.2.7839958

[6] Lara AR , and Schwarz MI . 2010 Diffuse alveolar hemorrhage. Chest 137:1164–1171. 20442117 10.1378/chest.08-2084

[7] de Prost N , Parrot A , Cuquemelle E , et al. 2012 Diffuse alveolar hemorrhage in immunocompetent patients: etiologies and prognosis revisited. Respir. Med. 106:1021–1032. 22541718 10.1016/j.rmed.2012.03.015

[8] Chung MP , Yi CA , Lee HY , et al. 2010 Imaging of pulmonary vasculitis. Radiology 255:322–341. 20413748 10.1148/radiol.10090105

[9] Lichtenberger JP 3rd , Digumarthy SR , Abbott GF , et al. 2014 Diffuse pulmonary hemorrhage: clues to the diagnosis. Curr. Probl. Diagn. Radiol. 43:128–139. 24791616 10.1067/j.cpradiol.2014.01.002

[10] Klimek M . 2019 Pulmonary lymphangitis carcinomatosis: systematic review and meta‐analysis of case reports, 1970‐2018. Postgrad. Med. 131:309–318. 30900501 10.1080/00325481.2019.1595982

[11] Light RW , and Hamm H . 1997 Malignant pleural effusion: would the real cause please stand up? Eur. Respir. J. 10:1701–1702. 9272907 10.1183/09031936.97.10081701

[12] Misra S , Chaturvedi A , Misra NC , et al. 2003 Carcinoma of the gallbladder. Lancet Oncol. 4:167–176. 12623362 10.1016/s1470-2045(03)01021-0

[13] Munk PL , Muller NL , Miller RR , et al. 1988 Pulmonary lymphangitic carcinomatosis: CT and pathologic findings. Radiology 166:705–709. 3340765 10.1148/radiology.166.3.3340765

[14] Levy H , Horak DA , and Lewis MI . 1988 The value of bronchial washings and bronchoalveolar lavage in the diagnosis of lymphangitic carcinomatosis. Chest 94:1028–1030. 3180853 10.1378/chest.94.5.1028

[15] Poletti V , Romagna M , Allen KA , et al. 1995 Bronchoalveolar lavage in the diagnosis of disseminated lung tumors. Acta Cytol. 39:472–477. 7762334

[16] De Lassence A , Fleury‐Feith J , Escudier E , et al. 1995 Alveolar hemorrhage. Diagnostic criteria and results in 194 immunocompromised hosts. Am. J. Respir. Crit. Care Med. 151:157–163. 7812547 10.1164/ajrccm.151.1.7812547

